# Comparative Evaluation of Allplex Respiratory Panels 1, 2, 3, and BioFire FilmArray Respiratory Panel for the Detection of Respiratory Infections

**DOI:** 10.3390/diagnostics12010009

**Published:** 2021-12-22

**Authors:** Harshad Lade, Jung-Min Kim, Yousun Chung, Minje Han, Eun-Kyung Mo, Jae-Seok Kim

**Affiliations:** 1 Department of Laboratory Medicine, Kangdong Sacred Heart Hospital, Hallym University College of Medicine, Seoul 05355, Korea; harshadlade@gmail.com (H.L.); jungmin510@gmail.com (J.-M.K.); yousun623@kdh.or.kr (Y.C.); mjhan@kdh.or.kr (M.H.); 2 Department of Internal Medicine, Uijeongbu Eulji Medical Center, Eulji University College of Medicine, Uijeongbu 11759, Korea; ekmopark@gmail.com

**Keywords:** respiratory infections, viruses, multiplex RT-PCR, respiratory panel, molecular diagnosis

## Abstract

Multiplex nucleic acid amplification assays that simultaneously detect multiple respiratory pathogens in a single nasopharyngeal swab (NPS) specimen are widely used for rapid clinical diagnostics. We evaluated Allplex Respiratory Panel (RP) 1, 2, 3, and the BioFire FilmArray RP assay for detecting respiratory pathogens from NPS specimens. In all, 181 NPS specimens obtained from patients suspected of having respiratory infections during the non-influenza season (August–December 2019) were included. The Allplex RP 1, 2, and 3 detected 154 samples positive for respiratory viruses, whereas the BioFire FilmArray detected viruses in 98 samples. Co-infection with two or more viruses was detected in 41 and 17 NPS specimens by Allplex RP and the BioFire FilmArray RP, respectively. For adenoviruses, Allplex RP 1 detected 31 specimens, compared to 34 by the BioFire FilmArray. In all, 64 NPS specimens were positive for human enterovirus (HEV) and human rhinovirus (HRV) on the Allplex RP, in contrast to 39 HEV/HRV on the BioFire FilmArray. The parainfluenza virus (PIV-1–4) detection rate differed between the two systems. Most discrepant results were observed for NPS specimens with high cycle threshold values obtained by Allplex RP. This study showed concordant performance of the Allplex RP 1, 2, 3, and the BioFire FilmArray RP for the simultaneous detection of multiple respiratory viruses.

## 1. Introduction

Acute respiratory infections (ARIs) are a common and major cause of illnesses frequently seen in children, the elderly, and immunocompromised patients, leading to hospitalization [1,2]. Several bacteria, fungi, and viruses can result in respiratory infections, but viruses have been the major etiology in ARIs; the most common viruses include human adenovirus (AdV), human coronavirus (229E, NL63, OC43, HKU1), influenza A virus (FluA), influenza B virus (FluB), human bocavirus 1/2/3/4 (HBoV), human enterovirus (HEV), human metapneumovirus (MPV), human rhinovirus (HRV), parainfluenza virus 1 (PIV-1), parainfluenza virus 2 (PIV-2), parainfluenza virus 3 (PIV-3), parainfluenza virus 4 (PIV-4), and respiratory syncytial virus A/B (RSV-A/B) [3,4]. ARI symptoms include headache, flu, cold, cough, nasal discharge, congestion, wheezing, shortness of breath, and myalgia. With ARI symptoms such as flu, cold, and sore throat are common to different pathogens, the accurate and rapid detection of an etiological agent is crucial for timely patient management, preventing the secondary spread of infection, and reducing hospital stays [5]. Consequently, rapid multiplex nucleic acid amplification assays have been developed and widely used to detect respiratory pathogens in nasopharyngeal samples [6]. Multiplex real-time polymerase chain reaction (RT-PCR) assays utilize nucleic acids from etiological agents as biomarkers and allow the rapid simultaneous detection of multiple respiratory pathogens in a single test with an easy sample-to-answer workflow [7,8,9,10,11].

For example, Seegene Allplex™ Respiratory Panel (RP) 1, 2, 3 (Seegene Inc., Songpa-gu, Seoul, Korea) and bioMérieux BioFire^®^ FilmArray^®^ RP (BioFire Diagnostics, Salt Lake City, UT, USA) use a multiplex PCR technology to simultaneously detect multiple respiratory pathogens in nasopharyngeal swab (NPS) specimens in a single test with high sensitivity and specificity [12,13,14]. Allplex RP 1, 2, and 3 comprises a one-step RT-PCR test based on multiple detection temperature (MuDT™) technology, which detects multiple analytes in a single fluorescence channel without melting curve analysis within a total time of 210 min [15]. The Allplex RP operates on the Seegene workflow, consisting of a module for nucleic acid extraction and PCR setup, a 96-well PCR thermocycler, and a computer for data analysis and interpretation of results. This assay also provides the cycle threshold (Ct) values of the positive analytes. The Allplex RP simultaneously tests 19 different viruses in a single nasopharyngeal sample; with the target including Panel 1: FluA, FluA-H1, FluA-H1pdm09, FluA-H3, FluB, RSV-A, and RSV-B; Panel 2: AdV, HEV, MPV, PIV-1, PIV-2, PIV-3, and PIV-4; Panel 3: HBoV, human coronavirus 229E, NL63, OC43, and HRV.

The BioFire FilmArray RP is a cartridge-based nested RT-PCR test that utilizes melting curve analysis for the simultaneous detection of 21 different respiratory pathogens (18 viruses and three bacteria) in a single nasopharyngeal sample. The viral targets detected by BioFire FilmArray RP include AdV, human coronavirus (HCoV 229E, NL63, OC43, and HKU1), MPV, FluA, FluA-H1, FluA-H3, FluA-H1-2009, FluB, PIV-1, PIV-2, PIV-3, PIV-4, HEV/HRV, and RSV-A/B. In addition, the panel can detect three bacteria that commonly cause respiratory tract infections, including *Bordetella pertussis* (BPP), *Chlamydophila pneumoniae* (CP), and *Mycoplasma pneumoniae* (MP). With integrated nucleic acid extraction, nested multiplex PCR amplification, detection, automated data analysis, and interpretation of results as detected or not detected, the BioFire RP assay requires a run time of approximately 45 min.

The Allplex RP 1, 2, 3, and BioFire FilmArray RP assays are varied in the formulation and thus may vary in analytical performance. However, a direct comparative evaluation of these multiplexed panels for detecting respiratory pathogens has not yet been reported. Therefore, this study aimed to comparatively evaluate the performance of Allplex RP 1, 2, 3, and BioFire FilmArray RP assays for the simultaneous detection of multiple respiratory viruses in clinical NPS specimens that had been submitted for the diagnosis of respiratory infections. The diagnostic performance of the RP assays was evaluated based on positive and negative agreement.

## 2. Materials and Methods

### 2.1. Clinical Specimens and Study Design

This study was conducted at Kangdong Sacred Heart Hospital, Seoul, Korea, during the non-influenza season (August 2019 to December 2019). A total of 181 NPS specimens were collected from patients suspected of having respiratory infection in a universal transport medium (*UTM™*) (Copan Italia S.p.a, Brescia, Italy). After the analysis of BiFire FilmArray RP, we stored the NPS specimens at −70 °C and performed the Allplex RP analysis. The results obtained from multiplexed panels were compared for: (i) positive or negative for each analyte, (ii) degree of agreement in the detection of identical analytes, and (iii) performance based on the relation between cycle threshold (Ct) values generated by the Allplex RP.

### 2.2. Allplex Respiratory Pathogens (RP) 1, 2, 3 Testing

The 300 μL of NPS sample (with 100 μL elute) was processed for nucleic acid extraction using microLAB NIMBUS IVD (Seegene Inc., Korea). The NIMBUS IVD consists of a single set of reagents for automated nucleic acid (DNA and RNA) extraction from NPS specimens. Each reaction mixture contained 8 µL of extracted nucleic acid and 17 µL of one-step RT-PCR master mix (5× RP MOM, 5 µL of RNase-free water, 5 µL of 5× real-time one-step buffer, and 2 µL of real-time one-step enzyme) at a final volume of 25 µL. Multiplex RT-PCR was performed using a CFX96™ Real-time PCR System (Bio-Rad Laboratories, Hercules, CA, USA). The results were analyzed automatically using Seegene Viewer V2.0 (Seegene Inc., Korea) and interpreted according to the manufacturer’s instructions. The entire process took approximately 210 min. Allplex RP 1, 2, and 3 allowed the simultaneous detection of 19 different viruses in a single NPS specimen. However, the *Mycoplasma pneumoniae* positive NPS specimens on the BioFire FilmArray RP assay were further tested using the Seegene PneumoBacter ACE Detection Panel (Seegene Inc., Korea), allowing the simultaneous detection of six respiratory bacterial pathogens in a single NPS specimen, including *Bordetella pertussis* (BP), *Chlamydophila pneumoniae* (CP), *Haemophilus influenzae* (HI), *Legionella pneumophila* (LP), *Mycoplasma pneumoniae* (MP), and *Streptococcus pneumoniae* (SP).

### 2.3. BioFire FilmArray RP Testing

The NPS sample (300 μL) was processed with the BioFire FilmArray RP according to the manufacturer’s instructions. The BioFire FilmArray RP integrates the following steps: (i) nucleic acid extraction: lysis by agitation and by sample buffer followed by purification of all nucleic acids using magnetic beads; (ii) nested multiplex PCR: first performs reverse transcription followed by multiplexed PCR1 and then performs multiple simultaneous PCR2 in the array to amplify sequences within PCR1 products; and (iii) interpretation of results: BioFire FilmArray software evaluates the endpoint melting curve data to detect target-specific amplicons and analyze it to generate a result for each analyte within a single NPS specimen. Possible results of each target in a valid run were reported as detected or not detected. The entire process took approximately 45 min for a single reaction (i.e., per specimen). The BioFire FilmArray RP allows the simultaneous detection of 21 different respiratory pathogens (18 viruses and three bacteria) in a single reaction.

## 3. Results

### 3.1. Comparative Evaluation of Multiplexed Panels

#### 3.1.1. Detection of Respiratory Viruses

Comparative evaluation of the Allplex RP 1, 2, and 3 against the BioFire FilmArray RP assay was performed for simultaneous detection of multiple respiratory viruses in 181 NPS specimens from patients suspected of respiratory infections. Coronavirus HKU1 and human bocavirus 1/2/3/4 (HBoV) could not be comparatively analyzed because these analytes were included in only one of the two systems. HEV and HRV have been reported separately by the Allplex RP 2 and 3 assays, whereas the BioFire FilmArray RP assay does not differentiate between them and reports as a single human rhinovirus/enterovirus (HRV/HEV). RSV-A and RSV-B were reported separately by the Allplex RP 1 assay, whereas the BioFire FilmArray RP assay reports them as a single RSV-A/RSV-B. The Allplex RP 1, 2, and 3 assays only generated Ct values of detected analytes, which were accepted as positive and utilized to compare the performance of the BioFire FilmArray RP for the detection of respiratory viruses. The difference in quantity (Ct value) of detected analytes was not compared because the BioFire FilmArray RP assay provided results as detected or not detected without Ct values.

**Figure 1:**
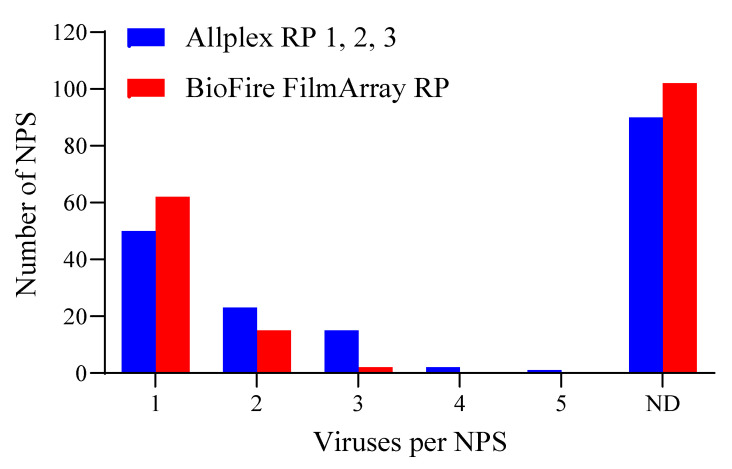
Comparison of the Allplex RP 1, 2, 3, and the BioFire FilmArray RP assays for the detection of respiratory viruses in nasopharyngeal swab (NPS) specimens (n = 181). ND: No virus detected.

Results of the 181 NPS specimens tested for respiratory viruses showed that Allplex RP 1, 2, and 3 assays detected one or more viral analytes per sample as: one virus (n = 50), two viruses (n = 23), three viruses (n = 15), four viruses (n = 2; sample no. 1 and 38), and five viruses (n = 1; sample no. 44) (Figure 1). The BioFire FilmArray RP detected one or more viral analytes per NPS specimen as one virus (n = 62), two viruses (n = 15), and three viruses (n = 2; sample no. 38 and 135). No viruses were detected in 90 NPS specimens by the Allplex RP 1, 2, and 3 assays and 102 NPS specimens using the BioFire FilmArray RP assay. However, several samples negative for a virus by one multiplexed panel were detected as positive by the comparator multiplexed panel. For example, the BioFire FilmArray RP detected NPS specimens positive for single viral infections (n = 5) were not detected by the Allplex RP 1, 2, and 3 assays. Seventeen of the 102 NPS specimens without viral infection by the BioFire FilmArray RP were detected positive for single or multiple viral infections by Allplex RP 1, 2, and 3 assays. In summary, among the 181 NPS specimens tested, the Allplex RP 1, 2, and 3 assays detected 154 samples positive for respiratory viruses, whereas the BioFire FilmArray RP detected viruses in 98 samples. Furthermore, co-infection of two or more viruses was detected in 41 and 17 NPS specimens by Allplex RP 1, 2, 3, and the BioFire FilmArray RP assays, respectively (Table S1). The simultaneously detected targets in the NPS specimens by Allplex RP 1, 2, 3, and the BioFire FilmArray RP assays are listed in Table S1.

The diagnostic performance of Allplex RP 1, 2, 3, and the BioFire FilmArray RP assays for detecting respiratory viruses in NPS specimens are summarized in Table 1. All 181 NPS specimens were found to be negative for coronavirus (229E, NL63, OC43, HKU1), influenza B virus (FluB), and human metapneumovirus (MPV). The Allplex RP 3 assay detected 10 NPS specimens positive for HBoV-1/2/3/4; however, this analyte was not included in the BioFire FilmArray RP and was not comparatively analyzed.

Complete agreement between the Allplex RP 1 and BioFire FilmArray RP was found for the detection of the FluA (n = 2; sample no. 116 and 135) (Table 1). No complete concordance was observed for AdV, HEV/HRV, PIV-1–4, and RSV-A/B. The most frequently detected viral analytes in NPS specimens were HEV/HRV, where Allplex RP 2 and 3 assays detected 24 HEV and 55 HRV, while the BioFire FilmArray RP assay detected 39 HEV/HRV-positive samples. All 39 HEV/HRV-positive NPS specimens by the BioFire FilmArray RP were also positive for HEV or HRV in the Allplex RP 2 and 3 assays. However, 12 HEV and 26 HRV-positive NPS specimens on the Allplex RP 2 and 3 were not detected by the BioFire FilmArray RP assay. For HEV/HRV detection, the best performance was achieved by the Allplex RP 2 and 3 (35.3%) compared to the BioFire FilmArray RP assay (21.5%). The NPS specimens positive for parainfluenza viruses on the Allplex RP 2 and BioFire FilmArray RP assays were PIV-1 (n = 6 and 4), PIV-2 (n = 5 and 2), PIV-3 (n = 5 and 6), and PIV-4 (n = 6 and 2). For comparison of individual RSV-A (n = 4) and RSV-B (n = 4) detected by Allplex RP 1 assay, the BioFire FilmArray RP agreed on seven positive NPS specimens. However, one RSV-A was only detected in the Allplex RP 1 assay, whereas only one RSV-A/RSV-B was detected in the BioFire FilmArray RP assay.

#### 3.1.2. Detection of *Mycoplasma Pneumoniae*

**Figure 2:**
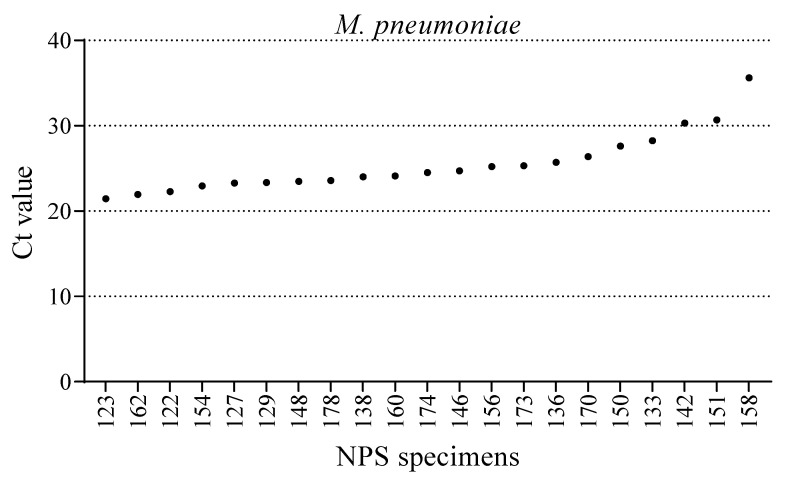
Cycle threshold (Ct) of *M. pneumoniae*-positive NPS specimens (n = 21) obtained from the Seegene PneumoBacter ACE Detection Panel. A complete concordance between the BioFire FilmArray RP and PneumoBacter ACE detection panel for the detection of *M. pneumoniae* was observed.

Compared with the Allplex RP 1, 2, and 3 assays, the BioFire FilmArray RP assay contains an additional respiratory target, that is, *M. pneumoniae*; thus, BioFire FilmArray RP results for this bacterium were accepted as correct. The NPS specimens positive for *M. pneumoniae* on the BioFire FilmArray RP were further confirmed by a separate Seegene PneumoBacter ACE detection panel, which also offers Ct values for the detected analytes. Twenty-one of the 61 NPS specimens were positive for *M. pneumoniae* in the BioFire FilmArray RP assay during the non-influenza epidemic (October and November 2019). Further evaluation of the same 61 NPS specimens on the PneumoBacter ACE detection panel showed complete concordance (n = 21) with the BioFire FilmArray RP assay. The PneumoBacter ACE detection panel generated Ct values of detected analytes, which were accepted as correct and utilized to compare the performance with the BioFire FilmArray RP. The Ct values obtained from the PneumoBacter ACE detection panel for *M. pneumoniae* ranged from 21.5–35.6 (Figure 2).

### 3.2. Discrepant Viral Analytes

Compared with the BioFire FilmArray RP, the only viruses detected by Allplex RP 1, 2, and 3 were AdV (n = 3), HEV/HRV (n = 25), PIV-1 (n = 3), PIV-2 (n = 3), PIV-3 (n = 1), PIV-4 (n = 4), and RSV-A (n = 1), which are shown in Table 1. In contrast, the only viruses detected by BioFire FilmArray RP assays over Allplex RP 1, 2, and 3 assays were AdV (n = 5), PIV-1 (n = 1), PIV-3 (n = 2), and RSV-A/RSV-B (n = 1). Compared to HEV/HRV detection by the BioFire FilmArray RP, the Allplex RP 2 and 3 assays include the differentiation of HEV and HRV. Among the 64 positive NPS specimens with the Allplex RP 2, 3 assays, 9 HEVs and 40 HRVs were positive individually, whereas the remaining 15 samples contained both HEV and HRV analytes (Table 1).

### 3.3. The Ct Value Comparision

**Figure 3:**
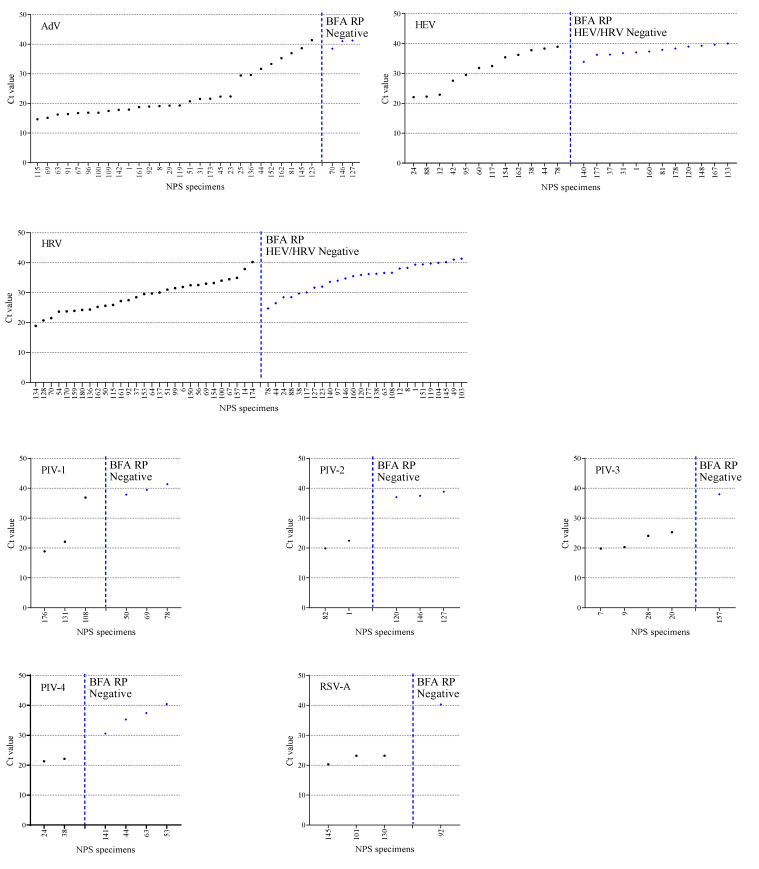
Cycle threshold (Ct) values of viruses detected in NPS specimens by the Allplex RP 1, 2, 3 assays. The Allplex RP assays generate Ct values of respiratory pathogens, which were accepted as correct and utilized to compare the performance against the BioFire FilmArray RP. The BioFire FilmArray RP assay does not differentiate between HEV and HRV as well as RSV-A, and RSV-B, and reports them as a single analyte HEV/HRV and RSV-A/RSV-B, respectively. The NPS specimens were ordered by ascending Ct values. Blue symbols denote NPS specimens positive on Allplex RP but negative on the BioFire FilmArray RP.

Discrepancies between the number of positive or negative viral analytes were estimated based on Ct values obtained from the Allplex RP 1, 2, and 3 assays. The Ct values obtained from Allplex RP 1, 2, and 3 assays spanned from 14.7 to 41.4 for AdV, 21.1 to 33.4 for FluA, 22.0 to 40.0 for HEV, 18.8 to 41.3 for HRV, 18.9 to 41.3 for PIV-1, 19.8 to 38.8 for PIV-2, 19.8 to 38.0 for PIV-3, 21.3 to 40.4 for PIV-4, and 17.3 to 40.2 for RSV-A/RSV-B (Figure 3). When comparing positive viral analytes on Allplex RP 1, 2, and 3 but negative on the BioFire FilmArray RP assay, most of the negative results corresponded to a low viral titer, as observed by high Ct values (>30.0). In this study, the Allplex RP 2 and 3 assays performance showed a higher detection rate for HEV (n = 24) and HRV (n = 55) than the HEV/HRV (n = 39) by the BioFire FilmArray RP. The 12 NPS specimens tested negative for HEV/HRV on the BioFire FilmArray RP assay had a Ct value >33.9 for HEV using the Allplex RP 2. However, among these 12 HEV/HRV-negative samples of the BioFire FilmArray RP, 5 NPS specimens (no. 1, 120, 140, 160, and 177) were additionally HRV-positive on the Allplex RP 3 assay. Furthermore, of the 55 HRV-positive NPS specimens on the Allplex RP 3 assay, 26 were HEV/HRV-negative by the BioFire FilmArray RP assay. Of these, five NPS specimens (nos. 24, 38, 44, 78, and 88) were had Ct values <30 for HRV using the Allplex RP 3 assay.

## 4. Discussion

Multiplexed panels that detect nucleic acids of viral or bacterial pathogens in a single test are being increasingly used for the diagnosis of multiple respiratory infections. However, such multiplexed panels often come at the expense of analytical performance [16]. This is crucial for the detection of ARIs because the pathogens can be present at low levels in clinical samples, and their abundance quickly drops over time despite symptoms being present [16]. This study evaluated the comparative performance of Allplex RP 1, 2, 3, and BioFire FilmArray RP assays to detect multiple respiratory viruses in clinical NPS specimens. The non-influenza season was selected for this study because influenza (FluA) abundance would be misleading for evaluating other respiratory viruses.

The results suggest that the Allplex RP 1, 2, and 3 assays detected a higher number of NPS specimens (n = 154) positive for respiratory viruses than the BioFire FilmArray RP assay (n = 98) from the 181 samples tested. It was not possible to compare the performance of the Allplex RP 1 and 2 against the BioFire FilmArray RP assays for human coronavirus, FluB, and MPV, as these analytes were not detected in any NPS specimens tested. Furthermore, a small number of FluA, PIV-1, 2, 3, 4, and RSV-A/B-positive NPS specimens were observed; however, they may not be sufficient for the comparative evaluation of multiplexed panels. The concordance between the Allplex RP 1 and BioFire FilmArray RP was 100% for FluA (n = 2). This complete concordance is most likely due to a smaller number of positive samples (1.1%), and the study period was during the non-influenza epidemic (October and November). However, the occurrence of concordant results with more positive samples could not be completely ruled out. Generally, the presently developed multiplex assays exhibit comparable performance in terms of sensitivity and specificity. For example, the previous evaluation of Allplex RP and the BioFire FilmArray RP to detect common respiratory viruses revealed a discordance of less than 10% [17,18,19].

The Allplex RP 2 and 3 assays detected 24 HEV and 55 HRV–positive NPS specimens versus the 39 HEV/HRV by the BioFire FilmArray RP assay. The Allplex RP assay enables specific identification of HEV and HRV, whereas the BioFire FilmArray RP reports them as a single HEV/HRV target. It should be noted that we did not use external HEV or HRV strains to check for specificity and cross-reactivity. However, differentiating HRV and HEV infections is crucial during outbreaks and epidemiology [20,21]. Discrepancies in HEV and HRV with HEV/HRV detection among multiplexed panels were analyzed based on the Ct values generated by Allplex RP 2 and 3, which were accepted as correct. The Ct value is inversely proportional to the amount of target nucleic acid and thus can be used as a relative indicator of pathogen load in the NPS specimen. The Allplex RP 2 and 3 assays generated Ct values for HEV and HRV, which were not detected as single HEV/HRV on the BioFire FilmArray RP range between 24.7–41.3. For example, five NPS specimens (no. 24, 38, 44, 78, and 88) were found HEV/HRV-negative in the BioFire FilmArray RP assay with Ct values <30 for HRV on the Allplex RP 3 (Figure 3). This suggests that despite the low Ct values of HRV in the Allplex RP 3 assay, the BioFire FilmArray RP could not detect HEV/HRV in the five NPS specimens. We visually checked the Allplex RP assay results for the Ct value and amplification curve; however, we could not determine the low amplification curve and positivity for the NPS specimens. Although we did not evaluate the diagnostic accuracy of Allplex RP for HEV or HRV detection with known strains, results from a previous study demonstrated that of the six HEV-positive NPS specimens on Allplex RP 2 assay, five results were confirmed positive by uniplex PCR and sequencing, except for one sample with a low viral load [22].

For adenovirus, Allplex RP 2 detected 31 NPS specimens versus 34 by the BioFire FilmArray assay. However, both the Allplex RP and BioFire FilmArray RP manufacturers did not provide information regarding the adenovirus genotypes. Discrepancies in the detection of AdV and PIV-2 by the Allplex RP 2 assay and BioFire FilmArray RP assay may be due to low viral loads. However, discrepancies in the detection of analytes due to low viral loads have not been confirmed with external controls. Furthermore, differences in the detection of analytes at higher viral titers (low Ct values) do not predict the sensitivity of multiplexed RP panels. For instance, the negative results for PIV-1 to 4 in some NPS specimens correspond to higher Ct values, suggesting post-infection or just carrier status. Hence, negative results by the BioFire FilmArray RP or positive results by Allplex RP for some NPS specimens should not be interpreted as an absence or presence of a viral analyte in clinical samples. In summary, Allplex RP 1, 2, and 3 showed higher sensitivity than the BioFire FilmArray RP in detecting the most common viruses associated with a respiratory infection. The performance of Allplex RP and the BioFire FilmArray RP assays for the detection of respiratory infections has been assessed in previous studies [13,22,23,24,25].

The detection of *M. pneumoniae* in several NPS specimens during the non-influenza epidemic suggests prominent seasonality. A complete concordance for *M. pneumoniae* (n = 21) positive results between the BioFire FilmArray RP and PneumoBacter ACE detection panel was observed. The main limitation of the Allplex RP assay is the absence of *M. pneumoniae* target, which was detected in 21 NPS specimens by BioFire FilmArray RP.

The multiplex RP assay has several advantages and disadvantages. The BioFire FilmArray RP, a cartridge-based testing method, can simplify the testing process and result in a shorter time. Allplex RP 1, 2, and 3 assays could be used for the high-throughput analysis of NPS specimens for respiratory viruses. Allplex RP assays can provide quantitative information about multiple viruses by analyzing individual Ct values, whereas the BioFire FilmArray RP is a qualitative test for the analytes in the NPS specimen. A negative RP result does not exclude the possibility of respiratory infection, and the negative test results may be due to the presence of sequence variants (or mutations) in the region targeted by the assay.

This study has some limitations: first, no quality control for molecular diagnostics (QCMD) or external viral controls have been tested using both RP assays. Second, in the case of discrepancies in results between Allplex RP 1, 2, and 3 and the BioFire FilmArray RP assays with more than two viruses present in one sample, a three-way comparison using a third-party assay has not been performed to verify the results. Third, no conclusion can be drawn to determine which of the two RP assays was correct in the case of discrepancies in positive analytes between the assays. Finally, the potential confounding factors were the primer sets used to amplify the selected targets by Allplex RP 1, 2, and 3 and the BioFire FilmArray RP. As this study was not designed to assess the sensitivity and specificity of RP assays, no conclusions have been drawn.

In summary, our data provide direct evidence that demonstrates differences between the Allplex RP and the BioFire FilmArray RP to detect respiratory pathogens. We provided a detailed comparison of the analytical performance of the two multiplexed RP panels and Ct value-based performance for viral analyte detection.

## 5. Conclusions

The Allplex RP 1, 2, and 3 assays and the BioFire FilmArray RP assay demonstrated concordant performance with some differences in the detection of respiratory viruses. In adenovirus, the BioFire FilmArray RP showed a higher detection rate than Allplex RP 2. However, the Allplex RP 2 and 3 assay showed higher sensitivity than the BioFire FilmArray RP for the detection of HEV and HRV. At lower viral loads, as observed by higher Ct values, the detection rate of the BioFire FilmArray RP was lower than that of the Allplex RP assay in general. The BioFire FilmArray RP assay is easy to perform and provides rapid detection of respiratory viruses. The main advantage of Allplex RP 1, 2, and 3 assays is the result of Ct values, which could help interpret results, although a standard measure of Ct value-based viral load may not be possible for the respiratory specimens.

## Figures and Tables

**Table 1:** Comparison of Allplex RP 1, 2, 3 and BioFire FilmArray RP assays for the detection of respiratory viruses in NPS specimens.

Viruses	Allplex RP 1, 2, and 3		BioFire FilmArray RP	
	Positive	Negative	Positive	Negative
AdV	31 (3)	150	34 (5)	147
229E	0	181	0	181
NL63	0	181	0	181
OC43	0	181	0	181
HKU1	NT	NT	0	181
FluA	2	179	2	179
FluB	0	181	0	181
HBoV-1/2/3/4	10	171	NT	NT
HEV/HRV ^a^	64 (25)	117	39	142
HEV	24 (12)	157	NT	NT
HRV	55 (26)	126	NT	NT
MPV	0	181	0	181
PIV-1	6 (3)	175	4 (1)	177
PIV-2	5 (3)	176	2	179
PIV-3	5 (1)	176	6 (2)	175
PIV-4	6 (4)	175	2	179
RSV-A/RSV-B ^b^	4 (1)/4	177/177	8 (1)	173
RSV-A	4 (1)	177	NT	NT
RSV-B	4	177	NT	NT

^a^: BioFire FilmArray RP assay does not differentiate between human enterovirus (HEV) and human rhinovirus (HRV) and reports them as a single analyte, HEV/HRV. ^b^: BioFire FilmArray RP assay does not differentiate between RSV-A and RSV-B and reports them as a single analyte RSV-A/RSV-B. Numbers in parentheses indicate the analytes detected only by the one RP assay. NT: not tested. Abbreviations: AdV, adenovirus; 229E, NL63, and OC43, coronavirus; FluA, influenza A virus; FluB, influenza B virus; HBoV, human bocavirus 1/2/3/4; HEV, human enterovirus; MPV, human metapneumovirus; HRV, human rhinovirus; PIV-1, PIV-2, PIV-3, and PIV-4, parainfluenza viruses 1, 2, 3, and 4; RSV-A and RSV-B, respiratory syncytial virus A and B.

## Data Availability

All data are available within this article.

## Supplementary Materials

The following are available online at https://www.mdpi.com/article/10.3390/diagnostics12010009/s1, Table S1. NPS specimens with co-infection of two or more viruses. The simultaneously detected targets by Allplex RP 1, 2, 3, and BioFire FilmArray RP assay are shown.
