# Supplementary material for: Comparative Evaluation of Allplex Respiratory Panels 1, 2, 3, and BioFire FilmArray Respiratory Panel for the Detection of Respiratory Infections
Source: Diagnostics (Basel). 2021 Dec 22;12(1):9. doi: 10.3390/diagnostics12010009 (PMC8775103; doi:10.3390/diagnostics12010009)
Supplement: Supplementary file 1 [file diagnostics-12-00009-s001.zip › diagnostics-1525817-supplementary.pdf]

**Table S1.** NPS specimens with co-infection of two or more viruses. The simultaneously detected targets by Allplex RP 1, 2, 3, and BioFire FilmArray RP assays are shown.

| NPS specimen no. | Allplex RP 1,2,3 | Ct value | Biofire FilmArray RP |
|---|---|---|---|
| 1 | AdV | Ct:17.93 | AdV |
| | PIV-2 | Ct:22.42 | PIV-2 |
| | HEV | Ct:37.04 | |
| | HRV | Ct:39.35 | |
| 8 | AdV | Ct:19.10 | AdV |
| | HRV | Ct:38.24 | |
| 9 | PIV-3 | Ct:20.29 | PIV-3 |
| | HBoV-1/2/3/4 | Ct:41.32 | |
| 12 | HBoV-1/2/3/4 | Ct:26.16 | |
| | HEV | Ct:22.87 | HEV/HRV |
| | HRV | Ct:38.03 | |
| 23 | AdV | Ct:22.35 | AdV |
| | HBoV-1/2/3/4 | Ct:41.74 | |
| 24 | PIV-4 | Ct:21.31 | PIV-4 |
| | HEV | Ct:22.05 | HEV/HRV |
| | HRV | Ct:28.46 | |
| 31 | AdV | Ct:21.54 | AdV |
| | HEV | Ct:36.81 | |
| 37 | HRV | Ct:28.45 | HEV/HRV |
| | HEV | Ct:36.31 | |
| 38 | HEV | Ct:37.77 | HEV/HRV |
| | HRV | Ct:29.7 | |
| | PIV-4 | Ct:22.13 | PIV-4 |
| | HBoV-1/2/3/4 | Ct:36.99 | PIV-3 |
| 44 | HEV | Ct:38.35 | HEV/HRV |
| | HRV | Ct:26.44 | |
| | AdV | Ct:31.68 | AdV |
| | HBoV-1/2/3/4 | Ct:32.39 | |
| | PIV-4 | Ct:35.23 | |
| 49 | HRV | Ct:41.01 | |
| | HBoV-1/2/3/4 | Ct:40.01 | |
| 50 | HRV | Ct:25.59 | HEV/HRV |
| | PIV-1 | Ct:37.84 | |
| 51 | AdV | Ct:20.75 | AdV |
| | HRV | Ct:30.99 | HEV/HRV |
| 63 | AdV | Ct:16.26 | AdV |

| | | | |
|---|---|---|---|
| | PIV-4<br>HRV | Ct:37.38<br>Ct:36.57 | |
| 67 | AdV<br>HRV | Ct:16.76<br>Ct:34.45 | AdV<br>HEV/HRV |
| 69 | AdV<br>HRV<br>PIV-1 | Ct:15.15<br>Ct:32.97<br>Ct:39.45 | AdV<br>HEV/HRV |
| 70 | AdV<br>HRV | Ct:38.49<br>Ct:21.46 | <br>HEV/HRV |
| 78 | HEV<br>HRV<br>PIV-1 | Ct:38.92<br>Ct:24.71<br>Ct:41.34 | HEV/HRV |
| 81 | AdV<br>HEV | Ct:36.96<br>Ct:37.87 | AdV |
| 88 | HEV<br>HRV | Ct:22.23<br>Ct:28.51 | HEV/HRV |
| 92 | AdV<br>HRV<br>RSV-A | Ct:18.97<br>Ct:27.46<br>Ct:40.29 | AdV<br>HEV/HRV |
| 100 | AdV<br>HRV<br>HBoV-1/2/3/4 | Ct:16.87<br>Ct:33.98<br>Ct:38.62 | AdV<br>HEV/HRV |
| 108 | PIV-1<br>RSV-B<br>HRV | Ct:36.86<br>Ct:29.77<br>Ct:36.64 | PIV-1<br>RSV-A/RSV-B |
| 115 | AdV<br>HRV<br>HBoV-1/2/3/4 | Ct:14.67<br>Ct:25.88<br>Ct:39.13 | AdV<br>HEV/HRV |
| 116 | FluA | Ct:26.76 | FluA (H1-2009) |
| | FluA (H1pdm09) | Ct:21.14 | |
| 117 | HEV<br>HRV<br>HBoV-1/2/3/4 | Ct:32.47<br>Ct:30.08<br>Ct:34.50 | HEV/HRV |
| 119 | AdV<br>HRV | Ct:19.33<br>Ct:39.72 | AdV |
| 120 | HEV<br>HRV<br>PIV-2 | Ct:38.97<br>Ct:35.90<br>Ct:37.00 | |
| 123 | HRV<br>AdV | Ct:32.01<br>Ct:41.38 | <br>AdV |

| | | | |
|---|---|---|---|
| 127 | PIV-2<br>HRV<br>AdV | Ct:38.84<br>Ct:31.65<br>Ct:41.25 | |
| 135 | FluA<br>FluA (H1pdm09) | Ct:33.42<br>Ct:30.35 | FluA<br>FluA (H1-2009)<br>AdV |
| 136 | AdV<br>HRV | Ct:29.63<br>Ct:24.35 | AdV<br>HEV/HRV |
| 140 | HRV<br>HEV | Ct:33.58<br>Ct:33.90 | |
| 143 | RSV-B | Ct:24.80 | RSV-A/RSV-B<br>AdV |
| 145 | HRV<br>RSV-A<br>AdV | Ct:40.20<br>Ct:20.29<br>Ct:38.63 | <br>RSV-A/RSV-B<br>AdV |
| 146 | PIV-2<br>HRV<br>AdV | Ct:37.44<br>Ct:34.76<br>Ct:41.02 | |
| 154 | HRV<br>HEV | Ct:33.20<br>Ct:35.39 | HEV/HRV |
| 157 | PIV-3<br>HRV | Ct:37.97<br>Ct:34.95 | <br>HEV/HRV |
| 160 | HRV<br>HEV | Ct:35.53<br>Ct:37.36 | |
| 161 | HRV<br>AdV | Ct:27.16<br>Ct:18.77 | HEV/HRV<br>AdV |
| 162 | HRV<br>HEV<br>AdV | Ct:25.23<br>Ct:36.22<br>Ct:35.26 | HEV/HRV<br><br>AdV |
| 177 | HRV<br>HEV | Ct:36.21<br>Ct:36.23 | <br><br>PIV-1 |

The red text indicates the targets that were only detected by one RP assay.
